# COPD Assessment Test Score Deterioration as a Predictor of Long-Term Outcomes in Patients Hospitalised for Chronic Obstructive Pulmonary Disease Exacerbation

**DOI:** 10.3390/jcm14041269

**Published:** 2025-02-14

**Authors:** Cristhian Alonso Correa-Gutiérrez, Zichen Ji, Irene Milagros Domínguez-Zabaleta, Manuel Delgado-Navarro, Ana López-de-Andrés, Rodrigo Jiménez-García, José Javier Zamorano-León, Luis Puente-Maestu, Javier de Miguel-Díez

**Affiliations:** 1 Respiratory Department, Gregorio Marañón General University Hospital, 28007 Madrid, Spain; cristhco@ucm.es (C.A.C.-G.); luis.puente@salud.madrid.org (L.P.-M.); javier.miguel@salud.madrid.org (J.d.M.-D.); 2 Faculty of Medicine, Complutense University of Madrid, 28040 Madrid, Spain; mandel04@ucm.es; 3 Gregorio Marañón Biomedical Research Institute, 28007 Madrid, Spain; 4 Respiratory Department, Infanta Leonor University Hospital, 28031 Madrid, Spain; irenemilagros.dominguez@salud.madrid.org; 5 Department of Public Health and Maternal & Child Health, Faculty of Medicine, Universidad Complutense de Madrid, 28040 Madrid, Spain; anailo04@ucm.es (A.L.-d.-A.); rodrijim@ucm.es (R.J.-G.); josejzam@ucm.es (J.J.Z.-L.)

**Keywords:** chronic obstructive pulmonary disease, disease exacerbation, healthcare resource utilisation, mortality

## Abstract

**Background:** While severe exacerbations are known to worsen the prognosis of patients with chronic obstructive pulmonary disease (COPD), the extent of this impact based on the degree of deterioration is unclear. COPD Assessment Test (CAT) scores increase during exacerbations, reflecting symptom worsening. This study aimed to compare healthcare resource utilisation and mortality among patients with COPD after a severe exacerbation, stratified by changes in CAT scores. **Methods:** This observational study included patients hospitalised for COPD exacerbation. The CAT questionnaire was administered twice: once referring to the time of admission and once to the stable phase. Patients were divided into tertiles based on symptom worsening. A prospective follow-up was conducted to compare emergency room visits, hospital admissions, and survival rates. **Results:** This study included 50 patients, of whom 30 (60%) were male. Their mean age was 70.5 years (standard deviation [SD]: 9.6), mean forced expiratory volume in the first second (FEV1) was 46.7% (SD: 0.8) of the predicted value, and median CAT score deterioration was 9 points (interquartile range: 5–15.25). Patients in the third tertile had earlier healthcare utilisation than those in the first tertile (emergency room visits: log-rank = 5.27, *p* = 0.022; hospitalisations: log-rank = 5.27, *p* = 0.022). Survival rates did not differ significantly among tertiles. **Conclusions:** Patients with greater CAT score deterioration experienced earlier COPD-related events, suggesting the need for closer monitoring after severe exacerbation.

## 1. Introduction

Chronic obstructive pulmonary disease (COPD) is characterised by episodes of acute worsening of respiratory symptoms, known as COPD exacerbations [1]. Severe exacerbations typically require hospitalisation for management [2].

During severe exacerbations, the degree of worsening may not be uniform, with symptoms worsening to varying extents depending on the aetiology, patient characteristics, and other circumstances [3,4]. Similarly, the impact on the patient’s quality of life is heterogeneous, depending on the degree of symptom worsening and their baseline status [5].

The COPD Assessment Test (CAT) is widely used to assess respiratory symptoms and health-related quality of life in patients with COPD [6]. This questionnaire consists of eight items: the first four pertain to respiratory symptoms (cough, sputum, chest tightness, and dyspnea), and the latter four address the disease’s impact on daily life (activities of daily living, confidence to leave the house, sleep quality, and energy) [6].

The CAT is typically administered to patients during the stable phase [7], although it has also been used during exacerbations in some studies [8,9]. By comparing CAT scores during the stable phase and an exacerbation, it is possible to determine the extent of deterioration in each patient for each exacerbation.

Exacerbations are a prognostic predictor in patients with COPD, with those with a history of prior exacerbations often exhibiting lower survival than those without such a history [10]. While it is well established that severe exacerbations are associated with worse outcomes, it is less clear whether the degree of symptom worsening during exacerbations influences subsequent patient outcomes.

This study aimed to describe patients’ clinical and functional characteristics and comorbidities according to tertiles of CAT score deterioration, analyse the number of emergency room visits and hospital admissions, compare the time to first emergency room visit and first hospitalisation, and compare survival during follow-up.

## 2. Materials and Methods

### 2.1. Study Design

This observational, single-centre, single-in-person-visit prospective cohort study included patients with COPD who were hospitalised for a severe COPD exacerbation as the primary diagnosis from September 2021 to March 2023, with prospective follow-up through medical record review after hospital discharge, which concluded in August 2024.

### 2.2. Study Population

This study enrolled patients based on the following inclusion and exclusion criteria. The inclusion criteria were (1) a known COPD diagnosis (post-bronchodilator spirometry with a ratio of the forced expiratory volume in the first second to the forced vital capacity [FEV1/FVC] < 0.70 in patients with respiratory symptoms and a history of smoking of >10 pack-years); (2) aged ≥40 years at study enrolment; and (3) able to understand and sign the informed consent document. The exclusion criteria were (1) known cognitive impairment or other neurological conditions that could limit the completion of the CAT questionnaire; (2) experiencing a COPD exacerbation in the two months prior to study enrolment, regardless of severity; and (3) a history of intensive care unit admission for a COPD exacerbation, irrespective of the time elapsed before study enrolment.

While all patients included in this study met all inclusion criteria and none of the exclusion criteria, not all eligible patients were enrolled due to the high healthcare workload caused by the COVID-19 pandemic during the patient enrolment period. To minimise selection bias, the sole criterion considered when deciding whether to enrol a patient was healthcare workload.

### 2.3. Data Collection

The variables were collected in two phases. Participants’ demographic, anthropometric, comorbidity, and clinical variables were collected at enrolment by reviewing medical records and patient interviews. At that point, the CAT questionnaire was administered to the participant twice, following an explanation and training to ensure it was completed in the manner specified for this study. First, the patient was asked to complete the CAT questionnaire, referring to the situation corresponding to the current hospital admission (CAT during exacerbation). Then, the patient was asked to complete the same questionnaire, referring to the situation during the two months before hospital admission (baseline CAT). The difference between CAT scores during exacerbation and baseline was calculated.

After discharge, participants’ medical records were periodically reviewed to collect data on clinical evolution and healthcare utilisation, including emergency room visits, hospital admissions, and mortality. If no new data were available in the medical record after hospital discharge, this information was obtained via telephone interviews with the participants.

### 2.4. Statistical Analysis

Categorical variables were presented as frequencies and percentages. The normality of the distribution of each continuous variable was assessed using histograms. Normally distributed continuous variables were presented as the mean and standard deviation (SD), and non-normally distributed continuous variables were presented as the median and interquartile range (IQR). Patients were stratified into tertiles of CAT score deterioration to create comparison groups with homogeneous sample sizes. Anthropometric, clinical, and functional characteristics were compared using Student’s *t*-test for continuous variables and Fisher’s exact test for categorical variables. Comparisons among the three tertiles were performed using omnibus tests, such as ANOVA or the Kruskal–Wallis test, depending on data normality. These tests assess differences across all groups simultaneously without conducting multiple pairwise comparisons. This approach was chosen to preserve statistical power and to minimise the risk of Type I errors associated with multiple tests. The events occurring during prospective follow-up were treated as time-dependent variables. These variables are presented using Kaplan–Meier curves and were compared using log-rank tests. Censoring was applied to the right, meaning that patients who did not experience the event of interest during the follow-up period were censored at their last known contact or at the end of the study period. Time-to-event analyses were conducted using Kaplan–Meier survival estimates, which appropriately account for right-censored data. A two-sided *p*-value of <0.05 was considered statistically significant for all comparisons. The Kaplan–Meier curves were generated using Stata (version 15; StataCorp LLC, College Station, TX, USA), while all other statistical analyses were conducted using SPSS (version 26; IBM Corp., Armonk, NY, USA).

### 2.5. Ethical Considerations

The study protocol was approved by the Research Ethics Committee of the Gregorio Marañón University General Hospital (approval code: 14/2019; approval date: 21 October 2019). Written informed consent was obtained from all participants before any study procedures.

## 3. Results

This study included 50 patients with a mean age of 70.5 years (SD: 9.6), of which 30 (60%) were male, and 8 (16%) were active smokers at study enrolment. Regarding their anthropometric data, the mean weight was 72.2 kg (SD: 15.9), the mean height was 1.63 m (SD: 0.08), and the mean body mass index (BMI) was 27.0 kg/m^2^ (SD: 5.6). Regarding their pulmonary function, the mean absolute FEV1 was 1.17 L (SD: 0.46), the mean FEV1 per cent predicted (FEV1pp) was 46.7% (SD: 0.8), the mean absolute FVC was 2.47 L (SD: 0.84), and the mean FVC per cent predicted (FVCpp) was 75.7% (SD: 18.4).

Regarding the CAT scores, the median baseline CAT score was 13.5 (IQR: 7–19), the median CAT score during exacerbation was 25 (IQR: 17.5–30), and the median difference between CAT scores during exacerbation and at baseline was 9 (IQR: 5–15.25). CAT scores differed significantly between exacerbation and baseline (*p* < 0.001). The median CAT score deterioration was 3 (IQR 2–5) in tertile 1, 9 (IQR 9–11.25) in tertile 2, and 19 (IQR 15.25–21.75) in tertile 3. The difference was statistically significant (*p* < 0.001).

Table 1 compares the anthropometric and functional characteristics among the tertiles of CAT score deterioration. The anthropometric and functional characteristics did not differ significantly among tertiles of CAT score deterioration.

Table 2 compares the presence of certain comorbidities and Charlson Comorbidity Index scores among the tertiles of CAT score deterioration. The prevalence of arterial hypertension was significantly higher in tertiles 2 and 3 (*p* = 0.032). However, Charlson Comorbidity Index scores did not differ significantly among the tertiles of CAT score deterioration.

The median follow-up period was 24.9 months (IQR: 17.4–28.4). During the follow-up, 14 patients (28.0%) died. Table 3 compares the number of events during follow-up according to the tertiles of CAT score deterioration. The number of emergency room visits or hospital admissions, either for any cause or due to COPD exacerbation, did not differ significantly among tertiles of CAT score deterioration. Table 4 compares the median time to event according to the tertiles of CAT score deterioration. Statistically significant differences were found in the median time to emergency room visits and hospital admissions, both for any cause and for COPD exacerbation, among tertiles of CAT score deterioration, whereas no significant differences were observed in the median time to death.

**Figure 1:**
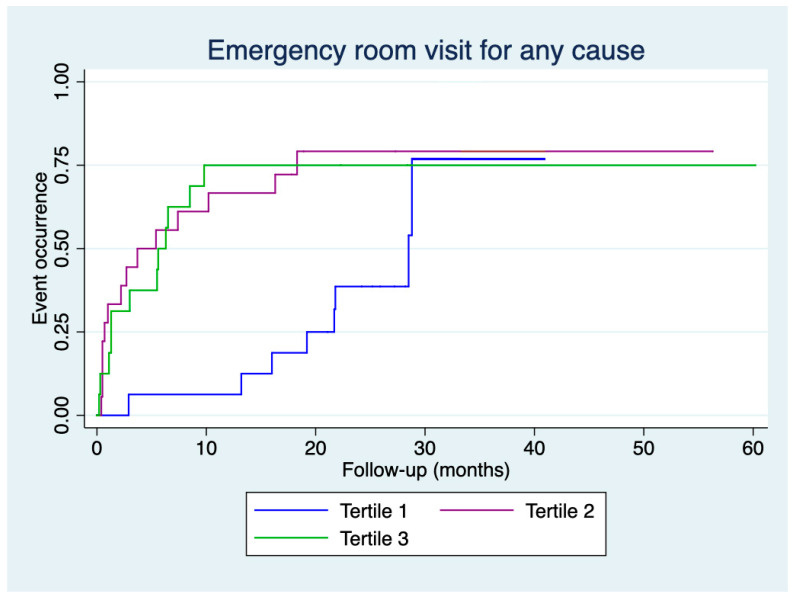
Time to the first emergency room visit for any cause according to tertiles of deterioration in CAT scores.

**Figure 2:**
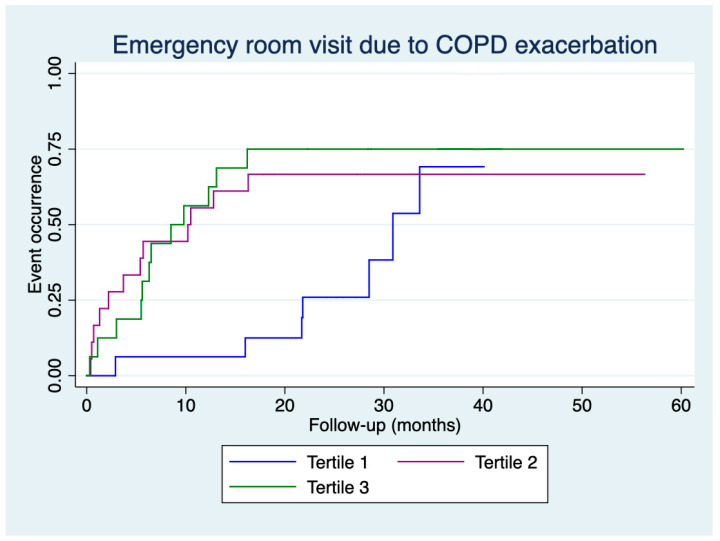
Time to the first emergency room visit for COPD exacerbation according to tertiles of deterioration in CAT scores.

Figure 1 and Figure 2 present the time to the first emergency room visit for any cause and COPD exacerbation, respectively, according to the tertiles of CAT score deterioration. An earlier emergency room visit for any cause was observed for patients in tertile 2 compared to those in tertile 1 (log-rank: 6.67, *p* = 0.010) and for patients in tertile 3 compared to those in tertile 1 (log-rank: 4.29, *p* = 0.038). Similarly, an earlier emergency room visit for COPD exacerbation was observed for patients in tertile 2 compared to those in tertile 1 (log-rank: 4.28, *p* = 0.038) and for patients in tertile 3 compared to those in tertile 1 (log-rank: 5.27, *p* = 0.022).

**Figure 3:**
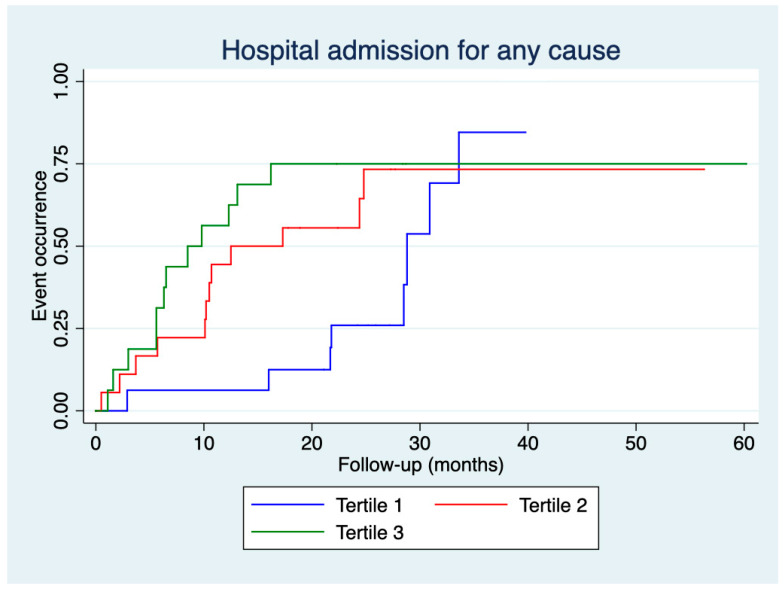
Time to the first hospital admission for any cause according to tertiles of deterioration in CAT scores.

**Figure 4:**
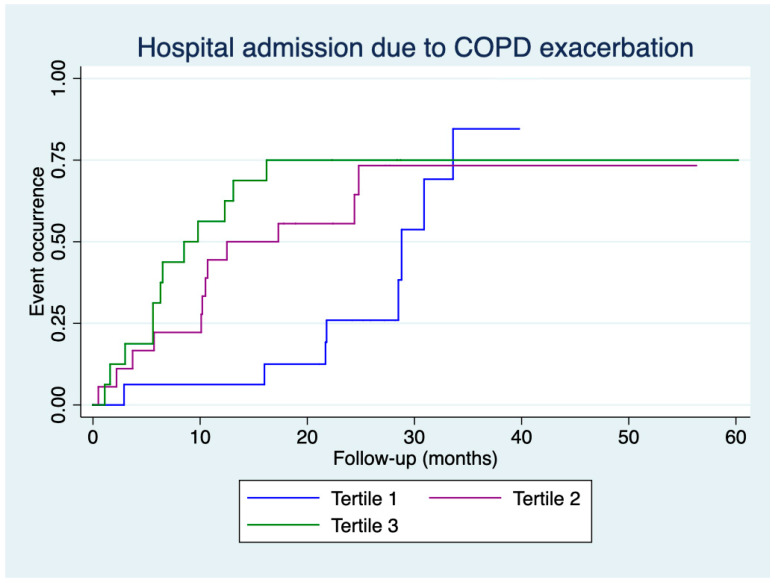
Time to the first hospital admission for COPD exacerbation according to tertiles of deterioration in CAT scores.

Figure 3 and Figure 4 present the time to first hospital admission for any cause and COPD exacerbation, respectively, according to the tertiles of CAT score deterioration. An earlier hospital admission for any cause was observed for patients in tertile 3 compared to those in tertile 1 (log-rank: 4.32, *p* = 0.038), and an earlier hospital admission for COPD exacerbation was observed for patients in tertile 3 compared to those in tertile 1 (log-rank: 5.27, *p* = 0.022).

**Figure 5:**
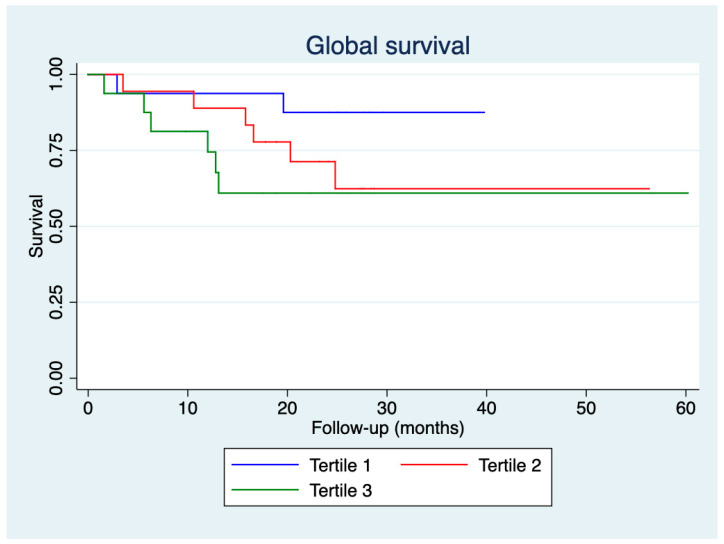
Patient survival according to tertiles of deterioration in CAT scores.

Figure 5 presents patient survival during follow-up according to the tertiles of CAT score deterioration. No statistically significant differences were observed.

## 4. Discussion

The primary finding of our study is that patients in the third tertile of CAT score deterioration experienced earlier emergency room visits and hospital admissions than those in the first tertile, with no differences observed in the number of events or survival.

The CAT questionnaire has high intra-patient sensitivity for detecting changes in health status [6,11], and it is commonly used in clinical practice to evaluate patient progress and even to detect exacerbations. It is frequently used to stratify the risk of patients with COPD [12] and to assess changes after interventions [13,14]. However, few studies have used variations in CAT scores to evaluate the severity of exacerbations [9].

In our study, the median decrease in CAT score during a COPD exacerbation requiring hospitalisation compared to the stable phase was 9 points. This finding highlights the utility of this questionnaire during exacerbations, as a change >2 points is typically considered clinically significant [15,16].

Previous studies have shown that CAT scores vary by COPD severity and comorbidities [12]. However, our study found no clinical, functional, or comorbidity differences among the tertiles of CAT score deterioration, except for a higher prevalence of arterial hypertension in patients in the third tertile. This observation simply reflects that the three groups were homogeneous regarding these characteristics, as the analysis focused on CAT score variation rather than absolute scores.

Our study observed no differences in the number of emergency room visits or hospital admissions, whether for any cause or COPD exacerbation, across the tertiles of CAT score deterioration. However, differences were found in the time to the event. Kaplan–Meier curves demonstrated that patients in the second and third tertiles had more events during the initial months of follow-up.

A relationship has been demonstrated between the severity of COPD exacerbations and the time to the next exacerbation, with patients experiencing severe COPD exacerbations having a shorter time to the next exacerbation than those with moderate exacerbations [17,18]. Similarly, patients with more severe COPD exacerbations have an increased risk of earlier non-respiratory incidents [19], including cardiovascular events [20,21]. This observation is attributed to the greater degree of symptom worsening and elevated levels of pro-inflammatory factors in patients with severe COPD exacerbations [2,22].

Our findings partially align with those of previous studies but also highlight the existence of different severities among patients with severe COPD exacerbations, which can be measured through CAT score deterioration.

Although patients in the third tertile of CAT score deterioration exhibited earlier ER visits and hospital admissions, no differences in overall survival were observed among the tertiles. This finding highlights the complexity of COPD outcomes, where short-term healthcare utilisation may not necessarily translate into differences in long-term mortality. Additionally, while CAT score deterioration reflects acute symptom burden, survival is influenced by a broader range of factors, including comorbidities, baseline functional status, and adherence to follow-up care. Nevertheless, multiple studies have demonstrated the negative impact of exacerbation severity on the survival of patients with COPD [5,23]. This discrepancy is likely due to the limited sample size of our study, resulting in insufficient statistical power to detect these differences. Future research with larger cohorts and longer follow-up periods could help clarify the relationship between acute exacerbation severity, as measured by CAT score changes, and long-term survival.

While differences in patient survival could not be demonstrated, it is known that COPD exacerbations entail significant healthcare [24] and social [25] costs. Therefore, closer monitoring of patients who experienced greater CAT score deterioration during a severe COPD exacerbation could potentially reduce healthcare-related costs. However, this hypothesis must be confirmed in specific studies.

Our study had several limitations. Firstly, its small sample size limits statistical power to detect small differences, which may have affected the survival comparison. This limitation is attributed to the high healthcare workload during the patient enrolment period due to the COVID-19 pandemic. Another consequence of the small sample size is that a stratified or multivariate analysis could not be performed, which may have left some confounding factors unidentified. Therefore, we recommend interpreting the results with caution. Secondly, administering the CAT questionnaire retrospectively for the stable phase may have introduced recall bias. The study design included a single in-person visit to avoid overburdening researchers and avoid patient travel to the hospital to complete the CAT questionnaire during the stable phase after recovering from the initial exacerbation. Future studies should include longitudinal CAT score measurements to better understand their relationship with outcomes. Thirdly, there may be some selection bias as not all eligible patients were enrolled; recruitment was restricted by the healthcare workload. Given these limitations, our results should be interpreted cautiously, acknowledging that this study is exploratory and its findings must be confirmed through larger studies.

## 5. Conclusions

Patients exhibiting greater CAT score deterioration during a severe COPD exacerbation are more likely to require earlier emergency room visits or hospitalisation. Therefore, these patients may require closer post-discharge monitoring to ensure better surveillance.

## Figures and Tables

**Table 1:** Comparison of anthropometric and functional characteristics according to tertiles of deterioration in CAT scores.

Variable	Tertile 1 (*n* = 16)	Tertile 2 (*n* = 18)	Tertile 3 (*n* = 16)	*p*-Value
Age, years (SD)	71.3 (7.9)	71.5 (9.9)	68.9 (11.0)	0.669
Male (%)	9 (56.3)	11 (61.1)	10 (62.5)	0.930
Active smoking (%)	3 (18.8)	2 (11.1)	3 (18.8)	0.779
Weight, kg (SD)	70.1 (19.6)	71.7 (15.5)	75.1 (12.2)	0.678
Height, m (SD)	1.64 (0.08)	1.63 (0.08)	1.65 (0.09)	0.743
BMI, kg/2 (SD)	25.9 (6.0)	27.2 (5.7)	27.9 (5.2)	0.620
FEV1, L (SD)	1.16 (0.50)	1.16 (0.50)	1.19 (0.39)	0.965
FEV1 pp % (SD)	46.0 (13.7)	47.6 (18.4)	46.5 (16.4)	0.957
FVC, L (SD)	2.39 (0.94)	2.38 (0.68)	2.67 (0.93)	0.574
FVC pp % (SD)	71.2 (17.1)	76.3 (19.5)	79.7 (18.4)	0.439

Abbreviations: SD: standard deviation; BMI: body mass index; FEV1: forced expiratory volume in the first second; FEV1 pp: forced expiratory volume in the first second per cent predicted; FVC: forced vital capacity; FVC pp: forced vital capacity per cent predicted.

**Table 2:** Comparison of the presence of certain comorbidities and Charlson Comorbidity Index scores according to tertiles of deterioration in CAT scores.

Comorbidity, *n* (%)	Tertile 1 (*n* = 16)	Tertile 2 (*n* = 18)	Tertile 3 (*n* = 16)	*p*-Value
Arterial hypertension	5 (31.3)	11 (64.7)	12 (75.0)	0.032
Dyslipidaemia	5 (31.3)	9 (52.9)	10 (62.5)	0.193
Diabetes mellitus	2 (12.5)	6 (35.3)	4 (25.0)	0.314
Atrial fibrillation	3 (18.8)	0 (0.0)	2 (12.5)	0.192
Ischemic heart disease	1 (6.3)	4 (23.5)	1 (6.3)	0.214
Heart failure	2 (12.5)	5 (29.4)	3 (18.8)	0.474
Obstructive sleep apnoea	3 (18.8)	3 (17.6)	2 (13.3)	0.913
Anxiety	0 (0.0)	2 (11.8)	0 (0.0)	0.141
Depression	1 (6.3)	3 (17.6)	2 (12.5)	0.607
Charlson Comorbidity Index, median (IQR)	4 (4–4)	2 (2–2)	1 (1–1)	0.562

Abbreviations: IQR: interquartile range.

**Table 3:** Comparison of the number of events during follow-up according to tertiles of deterioration in CAT scores.

Event, Median (IQR)	Tertile 1 (*n* = 16)	Tertile 2 (*n* = 18)	Tertile 3 (*n* = 16)	*p*-Value
Emergency room visits for any cause	2 (1–5.75)	2 (1–6)	2 (0–3.75)	0.651
Emergency room visit due to COPD exacerbation	1.5 (0.25–5.5)	1 (0–3.25)	1 (0–2)	0.413
Hospital admissions for any cause	1.5 (1–5.5)	1.5 (0–4)	1 (0–1.75)	0.070
Hospital admission due to COPD exacerbation	1.5 (0.25–5.5)	1 (0–3)	1 (0–1)	0.117
Death, *n* (%)	2 (12.5)	6 (33.3)	6 (37.5)	0.237

Abbreviations: IQR: interquartile range; COPD: chronic obstructive pulmonary disease.

**Table 4:** Comparison of the median time to event according to tertiles of deterioration in CAT scores.

Time to Event, Months (IQR)	Tertile 1 (*n* = 16)	Tertile 2 (*n* = 18)	Tertile 3 (*n* = 16)	*p*-Value
Emergency room visits for any cause	24.7 (19.7–28.4)	4.5 (0.7–17.9)	6 (1.3–19.2)	0.001
Emergency room visit due to COPD exacerbation	26.6 (21.7–30.3)	10.4 (2–21)	9.2 (5.5–20.8)	0.003
Hospital admissions for any cause	26.6 (21.7–28.7)	14.9 (9–24.5)	9.2 (5.6–20.8)	0.005
Hospital admission due to COPD exacerbation	26.6 (21.7–30.3)	15.1 (3.3–27.4)	9.2 (5.8–20.8)	0.007
Death	28 (24.4–36.9)	24.4 (17.5–27.7)	18.3 (10.4–27.8)	0.056

Abbreviations: IQR: interquartile range; COPD: chronic obstructive pulmonary disease.

## Data Availability

The data that support the findings of this study are available from the corresponding author upon reasonable request.
